# Reduced Trust in Bodily Sensations Predicts Suicidal Ideation in Hospitalized Patients With Major Depression: An Observational Study

**DOI:** 10.1111/sltb.70041

**Published:** 2025-08-21

**Authors:** Michael Eggart, Juan Valdés‐Stauber, Bruno Müller‐Oerlinghausen

**Affiliations:** ^1^ Center for Mental Health, Immanuel Hospital Rüdersdorf Brandenburg Medical School Theodor Fontane Rüdersdorf Germany; ^2^ Department of Psychiatry and Psychotherapy I Ulm University and Center for Psychiatry Südwürttemberg Ravensburg Germany; ^3^ Faculty of Medicine and Psychology Brandenburg Medical School Theodor Fontane Neuruppin Germany; ^4^ Charité – Universitätsmedizin Berlin Berlin Germany

**Keywords:** body awareness, interoception, major depressive disorder, outcome predictors, suicidal ideation, suicide prevention, trusting

## Abstract

**Background:**

Major depressive disorder (MDD) is associated with maladaptive self‐reported interoception, that is, abnormal bodily self‐experience. Although diminished body trust predicts suicidal ideation, interoceptive measures have not been considered in depressed inpatients, whose suicide risk regularly peaks post‐discharge. This study aims to explore interoceptive characteristics at admission that help identify inpatients at risk for suicidal ideation at discharge.

**Methods:**

The observational study included 87 depressed inpatients providing self‐ratings at both hospital admission (T0) and discharge (T1) on the BDI‐II and MAIA‐2. The statistical analysis included hierarchical logistic regression models and used ROC curve analysis to establish optimal cutpoints.

**Results:**

Suicidal ideation was found in 17.24% of patients at discharge, who reported lower baseline MAIA‐2 *Trusting* scores than non‐ideators (*p* = 0.01). Diminished *Trusting* (OR = 0.19), somatic comorbidity (OR = 16.77), and baseline suicidal ideation (OR = 24.01) significantly predicted suicidal ideation (T1). For *Trusting*, we estimated an optimal classification of subsequent suicidal ideation for the cutpoint ≤ 2.33 (AUC = 0.70 [95% CI 0.57, 0.83], sensitivity = 0.87, specificity = 0.44, positive predictive value = 0.25, negative predictive value = 0.94).

**Conclusions:**

Diminished body trust is a significant predictor for post‐treatment suicidal ideation in depressed inpatients. This finding emphasizes the importance of incorporating body‐centered approaches into multimodal treatment strategies, especially in inpatients under risk to prevent suicidal incidents.

## 1 Introduction

Major depressive disorder (MDD) is a common mental disorder associated with a substantial risk for suicide (Favril et al. 2023). Parallel to steadily increasing antidepressant prescription rates, mounting evidence indicates a concurrent rise in suicide rates, highlighting the urgent need for comprehensive and effective suicide prevention strategies (Amendola et al. 2024). Meta‐analytic findings raise serious doubts about the clinical significance of new‐generation antidepressants in preventing suicidal thoughts, suicide attempts, and suicides in routine psychiatric care (Braun et al. 2016; Hengartner et al. 2021). New‐generation antidepressants, such as selective serotonin reuptake inhibitors (SSRI), are even suspected of inducing ego‐dystonic suicidal thoughts, urges, and suicidal behaviors in rare cases (Hengartner et al. 2021; Stübner et al. 2018). Suicidal risk in patients with MDD typically tends to peak following the months after hospital discharge (Chung et al. 2017; Forte et al. 2019; Goldacre et al. 1993; Ho 2003; Krause et al. 2020; Park et al. 2013). This constitutes a significant obstacle to effective suicide prevention, as this period represents a critical window of heightened patient vulnerability outside of close clinical supervision and confrontation with own psychological challenges. Suicidal ideation—defined as “thoughts, ideas, or ruminations about the possibility of ending one's life, ranging from thinking that one would be better off dead to formulation of elaborate plans” (WHO 2024)—is a strong risk factor for suicidal mortality (Favril et al. 2023). However, there is currently a lack of reliable predictors (Lewitzka et al. 2017) that enable the early identification of individuals at heightened risk of suicidal ideation, particularly during the final phase of inpatient treatment. To improve prevention efforts, it is essential to detect such risk profiles as early as possible—ideally at the beginning of hospitalization. This underscores the need to explore novel clinical predictors of suicidal ideation from the outset of inpatient care. In this context, aberrant patterns of body awareness have been proposed as a potentially promising marker (Hielscher and Zopf 2021).

Interoception encompasses the sensation, interpretation, and integration of signals “originating from within the body, providing a moment‐by‐moment mapping of the body's internal landscape across conscious and unconscious levels” (Khalsa et al. 2018, 501). These internal sensations, ranging from heartbeat and respiration to hunger and temperature, primarily serve to maintain homeostatic and allostatic regulation (Tsakiris and Critchley 2016). The study of interoception has deep roots in phenomenology (concerning the anthropological dimension of embodiment), physiology (concerning the safeguarding of homeostasis), and neuroscience (including neurotransmitters, nerve pathways, and brain areas involved); its profound implications for mental health have recently gained significant attention (Cameron 2001; Craig 2002, 2009; Khalsa et al. 2018; Murphy et al. 2017; Tsakiris and Critchley 2016). For research purposes, the influential taxonomy by Garfinkel and Critchley (2013) classifies interoception into *three facets*, which are briefly described here and linked to psychopathological abnormalities, as a state of dysfunctional interoception is increasingly considered as a fundamental component of MDD (Eggart, Lange, et al. 2019; Harshaw 2015; Paulus and Stein 2010). First, patient's objective ability to accurately monitor their interoceptive states, known as *interoceptive accuracy* (Garfinkel et al. 2015), seems to be compromised in MDD. There is increasing evidence that affected subjects show diminished heartbeat perception accuracy (Eggart, Lange, et al. 2019) and reduced activation of the insular cortex during heartbeat perception tasks (Avery et al. 2014; Wiebking et al. 2010)—a state which potentially contributes to blunted positive affect intensity and decision‐making difficulties (Furman et al. 2013). Second, the subjective attention to bodily sensations, known as *interoceptive sensibility* or *self‐reported interoception* (Garfinkel and Critchley 2013; Mehling 2016), is abnormal in MDD. Core characteristics of MDD encompass altered pain perception (Thompson et al. 2016), increased somatic symptom burden (Kapfhammer 2006), anxiety‐driven attention styles directed toward unpleasant bodily cues (Flasinski et al. 2020; Zhou et al. 2024), emotion dysregulation linked with abnormal body awareness (Lyons, Strasser, et al. 2021; Zhou et al. 2022), and reduced trust in bodily sensations (Dunne et al. 2021). Third, depression has been associated with disturbed *interoceptive awareness*, characterized by a mismatch between interoceptive accuracy (objective perception of bodily signals) and interoceptive sensibility (subjective belief about those signals), resulting in prediction errors about interoceptive inputs (Paulus et al. 2019; Paulus and Stein 2010). Moreover, from a phenomenological perspective, patient's bodily feelings have been attributed to a “corporealization” of the personal significance of body‐related sensations (Fuchs 2005; Fuchs and Schlimme 2009), which is typified by sensations of constriction and oppression in the chest and abdomen, accompanied by feelings of alienation, heaviness, blockage, emptiness, paralysis, and passivity in the whole body (Lyons, Michaelsen, et al. 2021). These dysfunctional coenesthesias should not be solely construed as matters of well‐being, since abnormal self‐reported interoception has been recognized as a predictor for unfavorable treatment outcomes (Eggart et al. 2021; Eggart and Valdés‐Stauber 2021) and as a risk factor for residual symptoms of fatigue (Eggart et al. 2023a).

Recent studies have begun to shed light on the potential relationship between dysfunctional interoception and suicidality. Overall, a systematic review of the available evidence indicates that interoceptive impairments may be viable risk factors contributing to the emergence of suicidal ideation and behaviors (Hielscher and Zopf 2021). The literature consistently demonstrates a robust association between diminished trust in bodily sensations and the occurrence of suicidal ideation (Duffy et al. 2018, 2020; Forkmann et al. 2019; Gioia et al. 2022; Rogers et al. 2018). Additionally, low trust in interoceptive states is related to a history of suicide attempts (Duffy et al. 2018, 2020; Rogers et al. 2018). However, it is important to acknowledge the limitations present in most prior studies within this domain, particularly with regard to their use of cross‐sectional research designs and insufficient control for disease‐specific confounding variables such as depression severity (Hielscher and Zopf 2021). Furthermore, there is a notable lack of longitudinal studies that have specifically examined interoceptive predictors of suicidal ideation among inpatients diagnosed with MDD.

Therefore, we explored prospective associations between self‐reported interoception in MDD inpatients at admission and the likelihood of reporting suicidal ideation upon psychiatric discharge; aiming to enable early identification of at‐risk patients during the initial phase of hospital treatment. Furthermore, we aimed to delineate interoceptive baseline disparities in body trust between inpatient groups exhibiting differentiated change patterns in suicidal ideation from admission to discharge.

## 2 Materials and Methods

During all stages of the research process, the Declaration of Helsinki has been considered, and patients gave their written informed consent to participate in the study. The study was approved by the ethics committee of Ulm University (registration number: 13/17).

### 2.1 Procedure and Participants

This observational, naturalistic study included 87 participants who were consecutively admitted to a hospital ward specialized for the treatment of MDD in the Department of Psychiatry and Psychotherapy I of Ulm University (ZfP Südwürttemberg, Weissenau). Admitted patients were eligible to participate in the study if they met the research inclusion criteria (main diagnosis of major depression [ICD‐10 F32, F33], ≥ 18 years, proficiency of the German language). Patients were excluded from the study if they showed symptoms of psychosis, schizophrenia, substance abuse (reason for exclusion: psychotic states or excessive drug use can induce bodily hallucinations, which may disrupt interoceptive states [Kasten and Eilers 2023]), or intellectual disability (reason for exclusion: cognitive incapacity to participate in a questionnaire study). The therapy was not affected by participation in the study, and exclusion from the study had no consequences for further inpatient treatment. The treatment was provided in an open inpatient setting, not under coercion, for patients whose outpatient depression treatment was no longer adequate or for whom inpatient care was indicated due to elevated suicide risk. Included participants were 47.57 (±10.64) years old, 49 (56.32%) patients were female, and 60 (68.97%) were diagnosed with recurrent depressive disorder (F33). Most patients (*n* = 79, 90.81%) fulfilled criteria for severe depression (F3x.2); 8 (9.20%) patients were diagnosed with moderate depression (F3x.1). Diagnoses were assessed by trained psychiatrists or clinical psychologists according to ICD‐10 criteria (WHO 1992).

The present study is part of a larger project investigating interoceptive predictors of treatment outcomes in inpatients suffering from MDD. A detailed description of study characteristics, a study flow chart, and treatment components have been published in a previous paper (Eggart and Valdés‐Stauber 2021), but will be briefly summarized here. The treatment followed a guideline‐based approach and was not changed during the study period. All patients received psychotherapy (weekly sessions on the individual and group level), and most participants (*n* = 83, 95.40%) were treated with antidepressants. The antidepressant therapy was complemented by nursing interventions (e.g., crisis intervention, professional communication, relaxation techniques) and by exercise therapy. Study data were assessed within 48 h after admission (T0)/discharge (T1) to/from hospital, respectively. The median treatment duration was 8 weeks (interquartile range: 6.50–10.00). Somatic comorbidity was assessed and defined as the presence of physical illness concurrent with MDD and fulfilled the following criteria: (a) causes significant distress; (b) reduces the patient's quality of life; (c) is clearly objectively verifiable; (d) has a chronic character (lasting more than 6 months); (c) requires active medical treatment (medical, surgical, etc.); (d) requires regular monitoring (clinical, laboratory, imaging, etc.). In our sample, patients had various somatic comorbidities, including musculoskeletal disorders such as lower back pain, neck pain, spinal disc herniation, and spondylitis; neurological conditions such as migraine, multiple sclerosis, Parkinson's disease, polyneuropathy, and Willis‐Ekbom disease; dermatological conditions such as psoriasis; respiratory diseases such as chronic obstructive pulmonary disease (COPD) and sleep apnea; gastrointestinal disorders such as gastroesophageal reflux disease and ulcerative colitis; endocrine and metabolic conditions such as thyroiditis and diabetes mellitus; and cardiovascular conditions such as hypertension. Further details on participants' characteristics are presented in Table 1.

**TABLE 1:** Participant characteristics (*N* = 87).

Characteristics	Suicidal ideation: no (T1: *N* = 72)	Suicidal ideation: yes (T1: *N* = 15)	*t*	*χ* ^2^	df	*p*
	*M* ± SD/*N* (%)	*M* ± SD/*N* (%)				
Age (years)	47.21 ± 11.11	49.33 ± 8.13	−0.86	—	26.22	0.40
Sex (female)	41 (56.94%)	8 (53.33%)	—	0.00	1.00	1.00
BMI (kg/m^2^) (T0)	26.30 ± 4.99	27.19 ± 6.71	−0.49	—	17.37	0.63
School education						
≤ 9 years	13 (18.06%)	6 (40.00%)	—	4.74	2.00	0.09
10 years	33 (45.83%)	7 (46.67%)				
≥ 11 years	26 (36.11%)	2 (13.33%)				
Employment status						
Unemployed	14 (19.44%)	7 (46.67%)	—	5.12	2.00	0.08
Employed	53 (73.61%)	7 (46.67%)				
Retired	5 (6.94%)	1 (6.67%)				
Living alone	19 (26.39%)	7 (46.67%)	—	1.56	1.00	0.21
Main diagnosis (ICD‐10) (T0)						
F32 (single episode)	23 (31.94%)	4 (26.67%)	—	0.01	1.00	0.92
F33 (recurrent)	49 (68.06%)	11 (73.33%)				
BDI‐II (ex item 9) (T0)	28.82 ± 9.87	36.47 ± 8.83	−2.99	—	21.94	0.01*
Suicidal ideation (metric) (T0)	0.54 ± 0.63	1.20 ± 0.68	−3.47	—	19.34	0.00*
Suicidal ideation (dichot.) (T0)						
Yes	37 (51.39%)	1 (6.67%)	—	8.36	1.00	0.00*
No	35 (48.61%)	14 (93.33%)				
No. previous psychiatric inpatient treatments	0.99 ± 1.28	2.27 ± 2.60	−1.86	—	15.45	0.08
Somatic comorbidity	19 (26.39%)	8 (53.33%)	—	3.05	1.00	0.08
No. somatic comorbidities	0.99 ± 1.19	2.53 ± 2.13	−2.72	—	15.87	0.02*
No. psychotropic drugs (T0)	1.25 ± 1.20	2.00 ± 1.46	−1.89	—	18.09	0.08
Antidepressants (T1)						
SSRI	27 (37.50%)	3 (20.00%)	—	1.00	1.00	0.32
SNRI	20 (27.78%)	7 (46.67%)	—	1.28	1.00	0.26
TCA	15 (20.83%)	2 (13.33%)	—	0.10	1.00	0.76
NASSA	16 (22.22%)	2 (13.33%)	—	0.18	1.00	0.67
Treatment duration (weeks)	8.12 ± 3.22	10.80 ± 7.16	−1.42	—	15.20	0.18
MAIA‐2 (T0)						
Noticing	2.94 ± 1.00	2.78 ± 0.72	0.73	—	26.48	0.47
Not‐distracting	1.68 ± 0.77	1.64 ± 0.75	0.17	—	20.76	0.87
Not‐worrying	1.99 ± 0.93	2.24 ± 0.88	−1.00	—	21.10	0.33
Attention regulation	2.08 ± 1.01	1.90 ± 0.89	0.69	—	22.11	0.50
Emotional awareness	3.44 ± 1.10	2.96 ± 1.12	1.50	—	20.09	0.15
Self‐regulation	1.64 ± 0.94	1.77 ± 1.15	−0.41	—	18.12	0.68
Body listening	1.57 ± 0.99	1.51 ± 1.14	0.20	—	18.64	0.84
Trusting	2.31 ± 1.23	1.49 ± 0.93	2.92	—	25.29	0.01*

Abbreviations: %, relative frequency; BDI‐II, Beck Depression Inventory‐II; BMI, body mass index; df, degrees of freedom (Welch‐corrected for *t*‐tests); ICD‐10, International Statistical Classification of Diseases and Related Health Problems (10th revision); MAIA‐2, Multidimensional Assessment of Interoceptive Awareness, Version 2; *M ±* SD, mean ± standard deviation; *N*, absolute frequency; NASSA, noradrenergic and specific serotonergic antidepressants; SNRI, serotonin–norepinephrine reuptake inhibitors; SSRI, selective serotonin reuptake inhibitors; TCA, tricyclic antidepressants.

* *p* < 0.05 (two‐tailed).

### 2.2 Measures

#### 2.2.1 Multidimensional Assessment of Interoceptive Awareness, Version 2 (MAIA‐2)

The Multidimensional Assessment of Interoceptive Awareness, Version 2 (MAIA‐2) is a self‐rating scale which assesses differentiated aspects of self‐reported interoception (Eggart et al. 2021; Mehling et al. 2018). The multidimensional questionnaire includes 37 items which are rated on a 6‐point Likert scale (0 = “never”; 5 = “always”). The scales are averaged based on their respective items and are briefly described in the following (a sample item for each dimension and internal consistency reliability estimates for this study will be reported in brackets): *Noticing*—“awareness of uncomfortable, comfortable, and neutral body sensations” (McDonald's *ω* = 0.58; “I notice when I am uncomfortable in my body.”); *Not‐Distracting*—“tendency not to ignore or distract oneself from sensations of pain or discomfort” (*ω* = 0.67; “When I feel unpleasant body sensations, I occupy myself with something else so I don't have to feel them.”); *Not‐Worrying*—“tendency not to worry or experience emotional distress with sensations of pain or discomfort” (*ω* = 0.67; “When I feel physical pain, I become upset.”); *Attention Regulation*—“ability to sustain and control attention to body sensations” (*ω* = 0.87; “I can refocus my attention from thinking to sensing my body.”); *Emotional Awareness*—“awareness of the connection between body sensations and emotional state” (*ω* = 0.86; “I notice that my breathing becomes free and easy when I feel comfortable.”); *Self‐Regulation*—“ability to regulate distress by attention to body sensations” (*ω* = 0.76; “When I am caught up in thoughts, I can calm my mind by focusing on my body/breathing.”); *Body Listening*—“active listening to the body for insight” (*ω* = 0.77; “I listen for information from my body about my emotional state.”); *Trusting*—“experience of one's body as safe and trustworthy” (*ω* = 0.88; “I am at home in my body.”; “I feel my body is a safe place.”; “I trust my body sensations.”) (Eggart et al. 2021; Mehling et al. 2018). In previous research, the MAIA‐2 demonstrated adequate construct validity and—similar to our study—subthreshold internal consistency reliability on some scales (Mehling et al. 2012, 2018). The questionnaire also exhibited criterion validity in distinguishing between treatment response groups in depressed inpatients (Eggart et al. 2021). The strength of MAIA‐2 is its ability to differentiate between clinically beneficial and maladaptive interoceptive states by assessing attention styles toward the body that are anxiety‐driven or linked to self‐regulatory benefits (Mehling 2016). The questionnaire is frequently used in clinical samples and in the general population (Todd et al. 2020).

#### 2.2.2 Beck Depression Inventory‐II (BDI‐II)

The Beck Depression Inventory‐II (BDI‐II) is a frequently used self‐rating scale which assesses severity of depression in patients diagnosed with MDD. The questionnaire is unidimensional and includes 21 items that are rated on a 4‐point Likert scale (Hautzinger et al. 2006). A sample item of the BDI‐II is (Beck et al. 1996): “Loss of Pleasure” (0 = “I get as much pleasure as I ever did from the things I enjoy”; 1 = “I don't enjoy things as much as I used to”; 2 = “I get very little pleasure from the things I used to enjoy”; 3 = “I can't get any pleasure from the things I used to enjoy”). The items are summed up to assess overall depression severity. In the present study, we excluded item 9 from the sum score because this item was investigated as the outcome variable (please, see below). The internal consistency reliability of the BDI‐II (ex item 9) in the present study was good (*ω* = 0.89). In previous research, the measure demonstrated adequate reliability and validity (Beck et al. 1996; Hautzinger et al. 2006).

#### 2.2.3 Suicidal Ideation

Suicidal ideation was investigated by extracting item 9 from the BDI‐II. The exact wording of this item is as follows (Beck et al. 1996): “Suicidal Thoughts or Wishes” (0 = “I don't have any thoughts of killing myself”; 1 = “I have thoughts of killing myself, but I would not carry them out”; 2 = “I would like to kill myself”; 3 = “I would kill myself if I had the chance”). We dichotomized patients' responses to construct a binary variable which classified patients who reported suicidal thoughts of any severity (0 = “no suicidal ideation”; 1–3 = “suicidal ideation”).

### 2.3 Data Analysis

The statistical analysis was performed in R version 4.3.0 (R Core Team 2023) including the following R packages: cutpointr 1.1.1, dominanceanalysis 2.1.0, fmsb 0.7.5, ggpubr 0.6.0, margins 0.3.26.1, MBESS 4.8.1, OptimalCutpoints 1.1–5, performance 0.8.0, psych 2.1.9, and tidyverse 1.3.1. Missing data were minimized by requiring participants to complete all questionnaires—a patient (*n* = 1) with multiple missing data was excluded from the analysis. Patients lost to follow‐up (*n* = 22) were also excluded from the analysis—resulting in *n* = 87 completed and analyzed pre/post data. For all analyses, the significance level was a priori set to 5%. Baseline demographic, clinical, and interoceptive differences between patient groups with and without suicidal ideation were estimated by using *t*‐tests (metric variables) or Chi‐squared tests (categorial variables). We conducted a one‐way ANOVA, followed by Tukey's post hoc tests, to examine baseline differences on the MAIA‐2 *Trusting* subscale between inpatient groups showing distinct patterns of change in suicidal ideation from admission to discharge. This focus on the *Trusting* scale is based on its established relevance in suicidology research (Hielscher and Zopf 2021). A hierarchical logistic regression analysis was run to estimate the association between self‐reported interoception (T0) and suicidal ideation (T1). In the first block, the following variables were included to account for potential confounding effects with self‐reported interoception based on previous research (Eggart et al. 2023a; Eggart and Valdés‐Stauber 2021)—skewness (sk) and excess kurtosis (ku) for metric variables are reported in brackets: age (sk = −0.70; ku = −0.41), sex, body mass index (sk = 0.99; ku = 1.83), somatic comorbidity. The regression model was simultaneously adjusted for depression severity (BDI‐II ex item 9, T0, sk = 0.12; ku = −0.41) and suicidal ideation (T0) in the first block. In the second block of the analysis, we entered the MAIA‐2 subscales (Noticing: sk = −0.48; ku = −0.15; Not‐Distracting: sk = 0.43; ku = 0.08; Not‐Worrying: sk = 0.17; ku = 0.07; Attention Regulation: sk = 0.05; ku = −0.29; Emotional Awareness: sk = −0.78; ku = 0.19; Self‐Regulation: sk = 0.03; ku = −0.68; Body Listening: sk = 0.12; ku = −0.80; Trusting: sk = 0.19; ku = −0.38) to assess the predictive relevance of baseline self‐reported interoception. In a separate sensitivity analysis, we exclusively included the MAIA‐2 *Trusting* scale in the second block to determine its single contribution to the model. The odds ratio (OR) and average marginal effects (AME) were reported as effect measures along with 95% confidence intervals (95% CI). To assess the relative importance of each predictor—defined as its contribution to explaining variance in the criterion (i.e., suicidal ideation, t1)—we performed a dominance analysis, which evaluates the incremental contribution of predictors across all possible subset regression models (Azen and Traxel 2009).

We conducted a receiver operating characteristic (ROC) curve analysis to identify the diagnostic cutpoint at admission which predicts suicidal ideation at discharge. In the ROC curve analysis, a cutpoint for the MAIA‐2 *Trusting* scale (T0) was derived on the basis of Youden's index by maximizing the sum of sensitivity and specificity (Youden 1950). This cutpoint classified patients who reported suicidal ideation (=positive test) of any severity versus no suicidal ideation (=negative test) at the end of treatment. The area under the curve (AUC) was estimated by plotting the sensitivity (*y*‐axis) against 1 − specificity (*x*‐axis). The quality of classification, that is, accuracy, was further investigated by estimating the proportion of correctly classified cases. We also checked the assumptions of ROC curve analysis requiring a minimal correlation of |*r*| ≥ 0.30 for the associated metric variables (King 2011).

## 3 Results

Participants' characteristics grouped by suicidal ideation status (T1) are shown in Table 1. The prevalence of patients (*n* = 87) with any suicidal ideation was 56.32% (*n* = 49) at baseline (T0) and 17.24% (*n* = 15) at discharge (T1). Patients with suicidal ideation at psychiatric discharge exhibited greater baseline depression severity, more co‐occurring medical conditions, a higher burden of suicidal thoughts, and significantly lower scores on the MAIA‐2 *Trusting* subscale.

**FIGURE 1:**
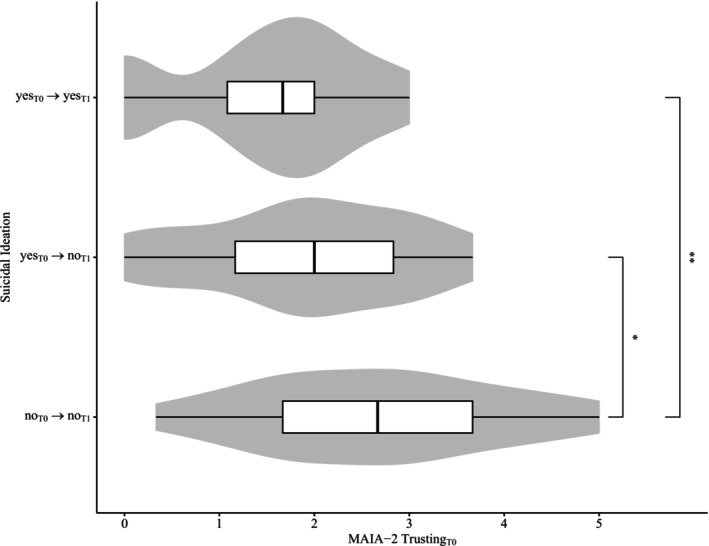
Differences concerning baseline body trust between groups with differentiated change patterns in suicidal ideation from admission (T0) to discharge (T1). Patients who reported no suicidal ideation at any time point showed the highest baseline ratings in body trust. The hybrid of a boxplot and a kernel density plot (gray) depicts the ratings on the MAIA‐2 *Trusting* scale. The white boxplots show the median (vertical black line) and first/third quarter (lower/upper limit of the box) of the data. Asterisks indicate statistically significant differences (Tukey HSD test: *p < 0.05, **p < 0.01).

Overall, suicidal ideation significantly decreased during the course of treatment (T0: 0.66 ± 0.68; T1: 0.18 ± 0.42), *t*(86) = 6.82, *p* < 0.01 (two‐tailed). In detail, suicidal ideation of any severity developed in 1 (1.15%) patient (“no_T0_ → yes_T1_”), disappeared in 35 (40.23%) patients (“yes_T0_ → no_T1_”), remained in 14 (16.09%) patients (“yes_T0_ → yes_T1_”), and was not reported at any time point by 37 (42.53%) patients (“no_T0_ → no_T1_”). We explored whether baseline scores on the MAIA‐2 *Trusting* scale differed between these groups after excluding the single patient who developed suicidal ideation during hospital treatment (Figure 1). There was a significant difference in *Trusting* between the three groups, *F*(2,83) = 7.01, *p* < 0.01, $\eta_{g}^{2}$ = 0.15 (large effect). The assumption of homoscedasticity was met by visually checking the Q–Q plot. In a post hoc multiple comparison analysis, the Tukey HSD test revealed that baseline *Trusting* scores in patients who stopped with suicidal ideation during treatment (“yes_T0_ → no_T1_”: 1.91 ± 1.08) were lower than in patients without any suicidal ideation at both time points (“no_T0_ → no_T1_”: 2.68 ± 1.26), *∆M* = −0.77 [95% CI −1.41, −0.13], *p*_adj_ = 0.01. Accordingly, patients in whom suicidal ideation remained unchanged during treatment reported lower baseline *Trusting* scores (“yes_T0_ → yes_T1_”: 1.50 ± 0.97) compared to the “no_T0_ → no_T1_” group, *∆M* = −1.18 [95% CI −2.04, −0.33], *p*_adj_ < 0.01. However, we found no significant difference between the “yes_T0_ → yes_T1_” and the “yes_T0_ → no_T1_” group, *∆M* = −0.41 [95% CI −1.28, 0.45], *p*_adj_ = 0.49.

In a logistic regression analysis (Table 2), we identified *Trusting*, AME = −0.13 [95% CI −0.21, −0.05], and the occurrence of somatic comorbidity, AME = 0.25 [95% CI 0.08, 0.41], as significant predictors of suicidal ideation over the time course, even after adjusting for baseline suicidal ideation that also showed a significant effect, AME = 0.20 [95% CI 0.08, 0.32]. Regarding self‐reported interoception, this means that a one‐unit increase on the MAIA‐2 *Trusting* scale at baseline is associated with an averaged 13.01% decreased probability of experiencing suicidal ideation at the time of discharge. The statistical model has significantly improved after the inclusion of the eight MAIA‐2 scales, showing an increase of explained variance by 23.23% (Nagelkerke's *R* ^2^), *χ* ^2^(8) = 16.83, *p* = 0.03. Assumptions of logistic regression analysis were checked for the analyzed models, and multicollinearity was not identified (VIF < 10).

**TABLE 2:** Prediction of suicidal ideation (T1) by baseline (T0) self‐reported interoception (*N* = 87).

Variable	Model 1					Model 2				
	*B*	SE	*z*	*p*	OR [95% CI]	*B*	SE	*z*	*p*	OR [95% CI]
Intercept	−6.79	2.67	−2.54	0.01*	0.00 [0.00, 0.14]	−11.87	5.02	−2.34	0.02*	0.00 [0.00, 0.04]
Age	0.02	0.04	0.57	0.57	1.02 [0.96, 1.10]	0.03	0.04	0.70	0.49	1.03 [0.95, 1.14]
Sex (ref.: female)	0.05	0.66	0.07	0.94	1.05 [0.28, 3.84]	1.62	1.00	1.63	0.10	5.08 [0.83, 45.81]
BMI	−0.01	0.07	−0.09	0.93	0.99 [0.87, 1.13]	0.01	0.08	0.07	0.94	1.01 [0.86, 1.18]
Som. Comorbidity	1.56	0.68	2.29	0.02*	4.76 [1.29, 19.39]	2.82	1.18	2.39	0.02*	16.77 [2.17, 266.74]
BDI‐II (ex item 9)	0.06	0.04	1.37	0.17	1.06 [0.98, 1.16]	0.10	0.05	1.83	0.07	1.10 [1.00, 1.23]
Suicidal ideation (T0)	2.44	1.15	2.13	0.03*	11.53 [1.72, 236.31]	3.18	1.52	2.09	0.04*	24.01 [2.20, 1527.26]
MAIA‐2 N	—	—	—	—	—	−0.47	0.75	−0.63	0.53	0.63 [0.13, 2.66]
MAIA‐2 ND	—	—	—	—	—	0.23	0.69	0.34	0.73	1.26 [0.29, 5.11]
MAIA‐2 NW	—	—	—	—	—	1.01	0.67	1.51	0.13	2.75 [0.80, 11.80]
MAIA‐2 AR	—	—	—	—	—	0.54	0.79	0.68	0.50	1.71 [0.39, 9.33]
MAIA‐2 EA	—	—	—	—	—	−0.08	0.59	−0.14	0.89	0.92 [0.26, 2.82]
MAIA‐2 SR	—	—	—	—	—	0.29	0.59	0.49	0.63	1.33 [0.42, 4.51]
MAIA‐2 BL	—	—	—	—	—	1.05	0.70	1.50	0.13	2.86 [0.83, 14.25]
MAIA‐2 T	—	—	—	—	—	−1.66	0.65	−2.57	0.01*	0.19 [0.04, 0.56]
Hosmer–Lemeshow goodness‐of‐fit test	*χ* ^2^(8) = 9.95, *p* = 0.27					*χ* ^2^(8) = 13.71, *p* = 0.09				
Akaike information criterion	73.96					73.13				
−2 log‐likelihood	59.96					43.13				
Nagelkerke's *R* ^2^	34.20%					57.43%				

Abbreviations: *B*, regression coefficient; BMI, body mass index (kg/m^2^); MAIA‐2, Multidimensional Assessment of Interoceptive Awareness, Version 2 (subscales: AR, attention regulation; BL, body listening; EA, emotional awareness; N, noticing; ND, not‐distracting; NW, not‐worrying; SR, self‐regulation; T, trusting); OR [95% CI], odds ratio [95% confidence interval]; SE, standard error; *z*, *z*‐test statistic.

* *p* < 0.05 (two‐tailed).

In an additional sensitivity analysis, we exclusively included the MAIA‐2 *Trusting* scale in the hierarchical regression analysis to estimate its single contribution to the model and found a comparable significant effect, *B* = −0.76, SE = 0.38, *z* = −1.99, *p* < 0.05, OR = 0.47 [95% CI 0.20, 0.94], AME = −0.08 [95% CI −0.15, −0.01]. The explained variance increased by 6.82%; *χ* ^2^(1) = 4.61, *p* = 0.03.

**FIGURE 2:**
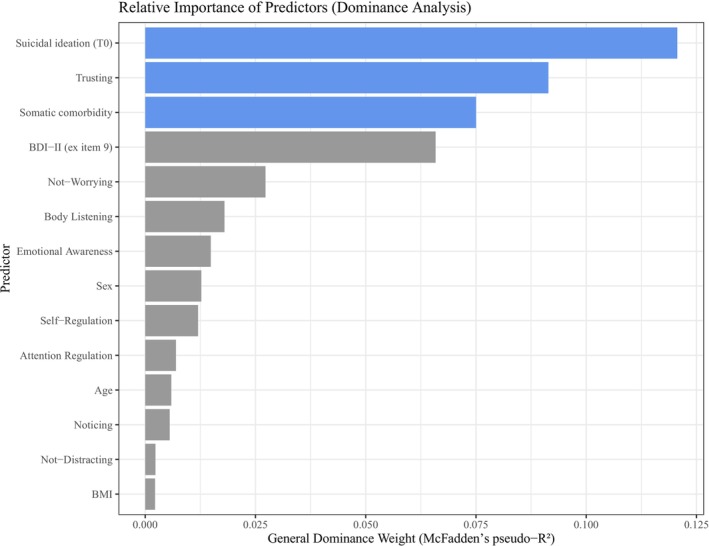
Relative predictor importance based on general dominance weights. The bar plot displays general dominance weights (McFadden's pseudo‐*R* ^2^) for each predictor from the logistic regression model. Predictors are ordered by their contribution to explained variance. Key predictors—suicidal ideation (T0), MAIA‐2 *Trusting*, and somatic comorbidity—are highlighted in blue. Higher dominance weights indicate greater relative importance in predicting suicidal ideation (T1).

We conducted a dominance analysis to evaluate the relative importance of predictors of the criterion suicidal ideation (T1). General dominance weights, based on McFadden's pseudo‐*R* ^2^, indicated that baseline suicidal ideation (0.121), MAIA‐2 *Trusting* (0.091), and somatic comorbidity (0.075) were the most influential predictors. Depression severity—although not significant—also contributed substantially (0.066), while other predictors showed comparatively smaller effects (Figure 2). The full model accounted for 46.07% of the variance in suicidal ideation (McFadden's pseudo‐*R* ^2^ = 0.46).

**FIGURE 3:**
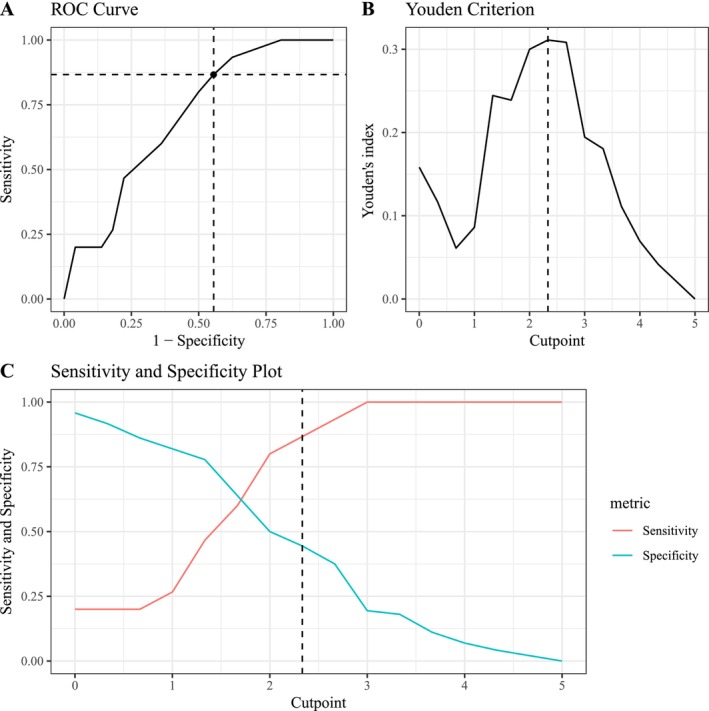
Results of ROC curve analysis establishing a binary classification of baseline MAIA‐2 *Trusting* scores which predict suicidal ideation at discharge. (A) The ROC curve shows the true‐positive rate (sensitivity, *y*‐axis) and the false‐positive rate (1 − specificity, *x*‐axis) for the optimal cutpoint (dashed line). (B) The optimal cutpoint was extracted following the Youden criterion (Youden 1950), ${\mathrm{YI}}_{c}=\max\left({\mathrm{Se}}_{c}+{\mathrm{Sp}}_{c}-1\right)=0.31$, which yielded the cutpoint *c* ≤ 2.33 (dashed line). There was no evidence of multiple cutpoints. (C) Sensitivity and specificity plot for every possible baseline *Trusting* cutpoint as a predictor of suicidal ideation. The dashed line shows the optimal cutpoint according to the Youden criterion.

The central assumption of ROC curve analysis was checked by showing a significant correlation between MAIA‐2 *Trusting* (T0) and suicidal ideation (T1), which slightly fell below the *r* > |0.30| criterion, *r* = −0.28 (95% CI −0.46, −0.08); *p* < 0.01. Regarding the total sample, a ROC cutpoint of ≤ 2.33 on the *Trusting* scale (Figure 3) optimally classified patients under subsequent risk for suicidal ideation at discharge (AUC: 0.70 [95% CI 0.57, 0.83]; sensitivity: 0.87 [95% CI 0.60, 0.98]; specificity: 0.44 [95% CI 0.33, 0.57]; accuracy: 0.52). In this highly depressed sample, the positive predictive value (PPV), that is true positive/(true positive + false positive) = 13/(13 + 40), was 24.53% [95% CI 16.50, 74.79]. The negative predictive value (NPV), that is, true negative/(true negative + false negative) = 32/(32 + 2), was 94.12% [95% CI 78.37, 96.31]. This means that, based on the prevalence of suicidal ideation (T1) = 17.24% in this sample, patients with a baseline *Trusting* score > 2.33 were at low risk for suicidal ideation, whereas every fourth patient with a score ≤ 2.33 showed suicidal ideation at discharge.

## 4 Discussion

In the present study, we sought to identify interoceptive predictors of suicidal ideation in patients suffering from MDD. In summary, low baseline scores on the MAIA‐2 *Trusting* subscale, which reflects experiencing the body as safe and trustworthy, significantly predicted suicidal ideation at psychiatric discharge. This association showed strong relative importance, independent of baseline depression severity, preexisting suicidal ideation, and somatic comorbidity. Notably, *Trusting* was the only interoceptive dimension that accurately discriminated between patients with and without suicidal ideation. Main findings and clinical implications for suicide prevention are discussed in the following sections.

Research on the prediction of suicidal ideation has so far yielded few conclusive results, particularly concerning prospective predictions over the time course of hospital treatment (Beck et al. 1989; Hawton et al. 2013). Our results underscore the clinically relevant role of interoceptive trust, captured by the MAIA‐2 *Trusting* subscale, in predicting suicidal ideation. *Trusting* emerged as the second most influential predictor, only surpassed by baseline suicidal ideation, and ranked ahead of other clinically salient variables such as somatic comorbidity and depression severity. Although the latter did not reach statistical significance, likely due to power limitations, the prominence of *Trusting* underscores the potential importance of bodily trust in shaping suicidal thoughts. This is further reflected in the comparable variance explained by *Trusting* and baseline suicidal ideation, highlighting its relevance as a crucial but underrecognized clinical marker. Strengthening bodily trust through interventions targeting interoceptive sensibility could offer a valuable, yet often overlooked pathway for suicide prevention (see detailed discussion below).

Moreover, we identified—for the first time—a clinical cutpoint on the *Trusting* subscale that significantly predicted patients with subsequent suicidal ideation, offering a potential diagnostic marker for early identification of at‐risk patients. The cutpoint demonstrated strong sensitivity, indicating that a substantial proportion of patients who reported suicidal ideation by the time of hospital discharge had a high probability of exhibiting subthreshold scores on the *Trusting* subscale at baseline. However, the specificity of this cutpoint was suboptimal, leading to a higher rate of false‐positive classifications by this diagnostic classifier. At first glance, the limitations of low specificity may seem disappointing, as it results in a low positive predictive value (PPV = 24.53%) in our sample. However, low PPVs are not uncommon even in established medical diagnostics. For instance, the PPV of mammography for breast cancer in women under age 50 can range between 16% and 26% (Peeters et al. 1987); PSA testing for prostate cancer yields PPVs of around 29% (Teoh et al. 2017); and D‐dimer testing for pulmonary embolism often shows PPVs as low as 17.5% (Abolfotouh et al. 2020). These examples illustrate that low PPVs do not necessarily undermine clinical utility—especially in early risk detection, where sensitivity and the ability to flag potentially critical cases take precedence over specificity (Pepe et al. 2016). Moreover, it is important to consider that—given the current scarcity of predictors for suicidal ideation, even a predictor with low PPV is a better choice than having no reliable predictors at all, which is currently the case in psychiatric care. The cutpoint may help to identify patients at risk for suicidal ideation, enabling clinicians to tailor treatments for this high‐risk group and better prepare for potential suicidal incidents in the critical period after hospital discharge—a time when suicidal risk is highest across all treatment stages (Chung et al. 2017; Forte et al. 2019; Goldacre et al. 1993; Ho 2003; Krause et al. 2020; Park et al. 2013). However, research indicates that suicidal ideation and progression from suicidal thoughts to suicidal attempt is a distinct phenomenon with different predictors and mechanisms. While factors such as depression, hopelessness, and impulsivity are significant predictors of suicidal ideation, these variables do not consistently differentiate between individuals with suicidal ideation and suicide attempters (Klonsky et al. 2016). In summary, our results underscore the relevance of interoceptive measures to predict suicidal ideation, particularly when baseline suicidal ideation data are unavailable or in populations reluctant to disclose such thoughts, while highlighting the need to overcome the measure's low specificity by considering further predictors in order to reduce false‐positive rates.

Our findings align with recent reports by Gioia et al. (2022), who examined interoceptive predictors of suicidal ideation in a sample of participants without a clinical diagnosis of MDD using a longitudinal follow‐up design. This study revealed that diminished body trust uniquely predicted both the presence and severity of suicidal ideation over a period of 6 months. However, unlike our study, the authors did not adjust for baseline depression severity. Furthermore, accumulating evidence from cross‐sectional studies demonstrates similar associations between appraising bodily sensations as unsafe or untrustworthy and the presence of suicidal thoughts, thereby further supporting our findings (Duffy et al. 2020; Hielscher and Zopf 2021; Perry et al. 2021; Rogers et al. 2018). Reduced body trust has been suggested as an indicator of detachment from the body, a state that may contribute to an intensified desire for death (Belanger et al. 2023). Therefore, Smith et al. (2021) sought to enhance interoceptive dimensions, particularly body trust, through an online intervention incorporating progressive muscle relaxation techniques, which effectively reduced outcomes related to suicidal ideation. These preliminary findings indicate that dysfunctional self‐reported interoception may function not only as a risk factor for suicidal ideation but also as a promising target for interventions aimed at reducing the risk of suicide attempts or completions. While causal dynamics remain challenging to establish, psychological frameworks such as the concept of the “body‐self” and the psychopathological construct of “depersonalization” warrant further exploration. Reduced body trust could be clinically reinterpreted as depersonalization, that is, a tendency not to take the bodily experience for granted but to focus on it perceptually, thereby awakening a gradual feeling of disconnection. Moreover, insights from philosophical and medical anthropology propose that experiential phenomena can be organized along fundamental pre‐reflective dimensions, including “corporality” (Fuchs 2018, 2021; Merleau‐Ponty 1945; Schmitz 2011; Valdés‐Stauber 2024). In the context of certain physical and mental disorders, disruptions in this dimension may contribute to distorted self‐perception and exacerbate psychopathological processes.

In hospitalized patients with MDD, it has been demonstrated that improvements in body trust during treatment‐as‐usual are associated with a better response to therapy—the effect was particularly pronounced in women (Eggart and Valdés‐Stauber 2021). However, clinicians may also be cognizant of emerging evidence indicating that antidepressants may exert adverse effects on interoceptive processing, including interoceptive numbing and diminished insular responsiveness to somatic signals (Broekaert et al. 2006; Cannon et al. 1994; Clouse et al. 1987; Gorelick et al. 1998; Livermore et al. 2024; Lyons, Strasser, et al. 2021; Peghini et al. 1998; Simmons et al. 2009; Zhou et al. 2024). These findings may be of critical importance for advancing the understanding of suicidal behavior, as blunted interoceptive processing (e.g., decreased awareness of bodily sensations and reduced insular activation during interoceptive tasks) distinguishes suicide attempters from non‐attempters (DeVille et al. 2020). Moreover, an experimental ketamine treatment in healthy individuals has demonstrated adverse effects on multiple facets of self‐reported interoception, notably inducing a state of disembodiment and significantly reducing MAIA‐2 *Trusting* scores (Kaldewaij et al. 2024). As a consequence, study participants expressed a strong desire for physical touch or contact from significant others. These preliminary findings may offer novel insights into mechanisms that support positive body awareness, which might be strengthened through increased skin‐to‐skin contact (Kaldewaij et al. 2024). Nevertheless, the notion that adjuvant administered touch‐based therapy might be appropriate as part of a comprehensive suicide prevention strategy remains highly speculative. Clinical studies have demonstrated that massage techniques based on *affective touch* (McGlone et al. 2014) can produce antidepressant, anxiolytic, analgesic, relaxing, and hopelessness‐reducing effects in individuals with MDD (Arnold et al. 2020; Baumgart et al. 2011, 2020; Hou et al. 2010; McGlone et al. 2024; Moyer et al. 2004; Müller‐Oerlinghausen et al. 2004; Papi et al. 2025)—possibly via interoceptive mechanisms (Bohlen et al. 2021; Eggart, Queri, and Müller‐Oerlinghausen 2019). However, there remains a significant lack of empirical research on the effectiveness of body‐oriented therapies in reducing suicidal ideation. Future investigations should address these gaps by exploring: (1) which interventions foster body trust, (2) which approaches help prevent suicidal ideation, attempts, or completed suicides, and (3) whether an interoceptive mechanism of action can be substantiated.

Additionally, somatic comorbidity emerged as a significant predictor of suicidal ideation, independent of baseline suicidal ideation and depression severity. Patients presenting with suicidal ideation at discharge also had a significantly higher individual number of physical illnesses. These findings are consistent with a growing body of research that links somatic illness such as cancer or pain with suicidal ideation and behavior (Stenager et al. 2021). In particular, chronic pain conditions such as migraine, back pain, arthritis, or fibromyalgia are associated with an elevated risk of suicidal ideation and attempts even after controlling for mental disorders (Stenager et al. 2021). Conversely, effective treatment of chronic pain is associated with a decreased risk of suicidal ideation (Kowal et al. 2014). It is therefore of uttermost importance to screen depressed patients for chronic pain conditions. However, also non‐painful conditions such as COPD or renal dysfunction are associated with higher odds of suicidal ideation (Goodwin 2011; Jhee et al. 2017; Sampaio et al. 2019). In summary, suicidal ideation is not solely a critical phenomenon in psychiatry but also a relevant concern among patients with somatic disorders (Stenager et al. 2021).

This study has several limitations that should be addressed in future research. The observational nature of this secondary data analysis limits definite causal interpretation, as it precludes control over potentially relevant and unknown confounders which interact with the MAIA‐2 *Trusting* scale. To strengthen the validity of these findings, future preregistered studies with larger sample sizes are needed—employing comprehensive designs, detailed multi‐item self‐report measures of suicidality, controlling for history of suicidal behavior, and especially including follow‐up assessments to predict future suicidal ideation/behavior. Larger sample sizes would also enhance control over type II errors, particularly for interoceptive predictors. Furthermore, our reliance on a psychiatric inpatient sample introduces a selection bias, restricting the generalizability of the findings to other populations. However, this homogeneous sample also represents a strength, as it allows us to estimate validated cutpoints relevant to typical psychiatric hospital settings, enhancing risk assessment in this population. Due to the exploratory nature of this study, we were unable to identify the underlying etiology of reduced body trust in MDD, an area that warrants further investigation (Eggart et al. 2023b). Additionally, the study was underpowered to explore potential interactions between somatic comorbidity and low MAIA‐2 *Trusting* scores, meaning that we cannot exclude the possibility that the observed association between *Trusting* and suicidal ideation may be specific to patients with somatic comorbidities. Despite the aforementioned limitations, this study has the advantage of generating hypotheses that open a new direction for research into the phenomenon of suicidal ideation.

## 5 Conclusion

Beyond preexisting suicidal ideation and the presence of somatic comorbidity, the level of self‐reported body trust at the onset of hospital treatment emerged as a significant and important predictor of suicidal ideation at discharge. Routinely assessing interoceptive trust in patients with depression could thus provide healthcare providers with valuable insights, facilitating the identification of individuals at elevated risk for suicidal ideation. If evidence of reduced body trust is present, the administration of antidepressants or ketamine should be approached with heightened caution as these substances can potentially induce both suicidal thoughts/behaviors in rare cases and further aggravate interoceptive impairments. Investigating the mechanisms by which interoception influences mental health outcomes may reveal promising therapeutic pathways, particularly by integrating psychological *and* body‐centered treatments into patient care to reduce suicide risk. Therefore, trust in interoceptive states may serve not only as an important prognostic factor but also as a potential target for interoceptive therapies aimed at supporting suicide prevention.

## Author Contributions

**Michael Eggart:** conceptualization (equal), data curation (equal), formal analysis (equal), investigation (equal), methodology (equal), resources (equal), software (equal), validation (equal), visualization (equal), writing – original draft (equal), writing – review and editing (equal). **Juan Valdés‐Stauber:** supervision (equal), validation (equal), writing – review and editing (equal). **Bruno Müller‐Oerlinghausen:** conceptualization (equal), supervision (equal), writing – original draft (equal), writing – review and editing (equal).

## Ethics Statement

The study was approved by the ethics committee of Ulm University (registration number: 13/17). The principles of the Declaration of Helsinki were followed.

## Consent

Patients gave their written informed consent.

## Conflicts of Interest

The authors declare no conflicts of interest.

## Data Availability

The datasets used and/or analyzed during the current study are available from the corresponding author on reasonable request.
